# HOXC9 directly regulates distinct sets of genes to coordinate diverse cellular processes during neuronal differentiation

**DOI:** 10.1186/1471-2164-14-830

**Published:** 2013-11-25

**Authors:** Xiangwei Wang, Jeong-Hyeon Choi, Jane Ding, Liqun Yang, Lambert C Ngoka, Eun J Lee, Yunhong Zha, Ling Mao, Bilian Jin, Mingqiang Ren, John Cowell, Shuang Huang, Huidong Shi, Hongjuan Cui, Han-Fei Ding

**Affiliations:** 1 Department of Urology, Second Affiliated Hospital, Third Military Medical University, Chongqing, China; 2 Cancer Center, Georgia Regents University, Augusta, GA 30912, USA; 3 Department of Biostatistics and Epidemiology, Medical College of Georgia, Georgia Regents University, Augusta, GA 30912, USA; 4 Department of Pathology, Medical College of Georgia, Georgia Regents University, Augusta, GA 30912, USA; 5 Department of Biochemistry and Molecular Biology, Medical College of Georgia, Georgia Regents University, Augusta, GA 30912, USA; 6 State Key Laboratory of Silkworm Genome Biology, Institute of Sericulture and System Biology, Southwest University, Chongqing, China; 7 Department of Neurology, First Hospital of Yichang, Three Gorges University College of Medicine, Yichang, China; 8 Department of Neurology, Union Hospital, Tongji Medical College, Huazhong University of Science and Technology, Wuhan, China

**Keywords:** Neuronal differentiation, Cell cycle arrest, DNA damage response, E2F6, HOXC9, Neuroblastoma

## Abstract

**Background:**

Cellular differentiation is characterized by the acquisition of specialized structures and functions, cell cycle exit, and global attenuation of the DNA damage response. It is largely unknown how these diverse cellular events are coordinated at the molecular level during differentiation. We addressed this question in a model system of neuroblastoma cell differentiation induced by HOXC9.

**Results:**

We conducted a genome-wide analysis of the HOXC9-induced neuronal differentiation program. Microarray gene expression profiling revealed that HOXC9-induced differentiation was associated with transcriptional regulation of 2,370 genes, characterized by global upregulation of neuronal genes and downregulation of cell cycle and DNA repair genes. Remarkably, genome-wide mapping by ChIP-seq demonstrated that HOXC9 bound to 40% of these genes, including a large number of genes involved in neuronal differentiation, cell cycle progression and the DNA damage response. Moreover, we showed that HOXC9 interacted with the transcriptional repressor E2F6 and recruited it to the promoters of cell cycle genes for repressing their expression.

**Conclusions:**

Our results demonstrate that HOXC9 coordinates diverse cellular processes associated with differentiation by directly activating and repressing the transcription of distinct sets of genes.

## Background

Cellular differentiation is an essential process of normal development by which a stem or progenitor cell becomes a post-mitotic, specialized cell with unique morphology and function. In addition, it has long been recognized that differentiated cells of both normal and tumor origin are defective in the DNA damage response and repair at the global level, displaying a marked increase in sensitivity to ionizing radiation and other DNA damaging agents [1-3]. Consistent with these observations, recent studies have shown that brain and breast cancer stem cells, a small subpopulation of tumor cells thought to be responsible for initiating and sustaining tumor growth [4-6], are more resistant to irradiation and chemotherapy than bulk tumor cells [7-10]. Particularly interesting is the observation that inhibition of DNA damage checkpoint kinases can reverse the radioresistance of glioma stem cells [7]. Thus, a molecular understanding of cellular differentiation may suggest new therapeutic strategies that target both cell proliferation and the DNA damage response.

Among the genes that have a critical role in the control of cellular differentiation are the *HOX* gene family members. *HOX* genes encode a family of transcription factors that function as master regulators of morphogenesis and cell fate specification [11-13]. Dysregulation of *HOX* gene expression has been implicated in the pathogenesis of cancers of different tissue types. In most tumor types, *HOX* genes function as oncogenes to promote cancer development such as *HOXA9* in leukemia and *HOXB13* in ovarian and breast cancers [13,14]. However, in neuroblastoma, a common childhood malignant tumor of the sympathetic nervous system [15,16], there is evidence suggesting that *HOX* genes may function as tumor suppressors [13]. Particularly, downregulation of HOXC9 expression is significantly associated with poor prognosis in neuroblastoma patients [17,18].

Neuroblastoma cells can be induced to undergo neuronal differentiation by serum deprivation [19], nerve growth factor [20] or retinoic acid (RA) [21]. RA-induced neuronal differentiation of neuroblastoma cells is a well-established model for molecular investigation of neuronal differentiation [22]. We recently reported that RA-induced differentiation of neuroblastoma cells required the activation of several *HOX* genes [18,23]. Among them, HOXC9 appeared to be a major mediator of RA action in neuroblastoma cells. HOXC9 expression was upregulated by RA, and silencing HOXC9 expression conferred resistance to RA-induced differentiation. Importantly, ectopic HOXC9 expression alone was sufficient to induce growth arrest and morphologic differentiation in neuroblastoma cells, fully recapitulating the neuronal differentiation phenotype induced by RA [18].

Differentiated neuroblastoma cells morphologically and functionally resemble mature peripheral neurons characterized by G1 arrest, extensive neurite outgrowth, and significant resting potential. It has long been observed that differentiated neuroblastoma cells are highly sensitive to UV and X-ray radiation with a significantly reduced rate of DNA damage repair [20,24-27]. The molecular basis for the differentiation-induced radiosensitivity is not well understood. The biological functions of RA are mediated by multiple isotypes of RA receptors (RARs) and retinoid X receptors (RXRs), which form RAR/RXR heterodimers that bind RA response elements in the regulatory regions of RA target genes and regulate their transcription [28]. The complexity of multiple RARs and RXRs involved in the action of RA presents a daunting challenge to dissect the molecular mechanism that coordinates the diverse cellular events associated with differentiation. Thus, the finding that HOXC9 alone is able to initiate a robust transcriptional program that drives neuronal differentiation provides a unique experimental system for this investigation. In this study, we conducted genome-wide profiling of the HOXC9-initiated transcriptional program. Our investigation reveals that HOXC9 directly regulates the expression of three major sets of genes that separately control neuronal differentiation, cell cycle progression, and the DNA damage response.

## Results

### Gene expression profiling of HOXC9-induced neuronal differentiation

**Figure 1:**
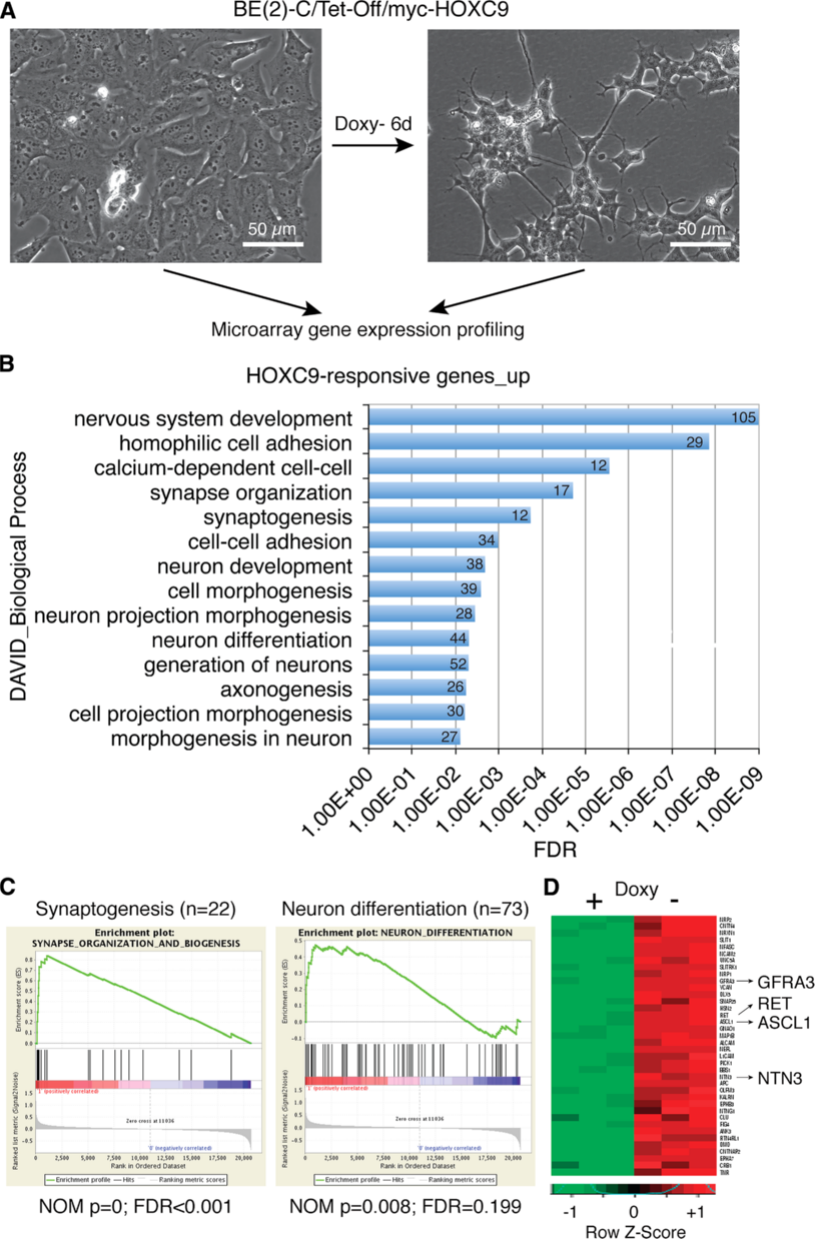
**Global upregulation of neuronal genes by HOXC9. (A)** Schematic of experiment. Doxy, doxycycline. **(B)** DAVID analysis of upregulated HOXC9-responsive genes for enriched GO biological process categories (enrichment fold > 2.0; FDR <1%). The number of genes for each biological process category is indicated. **(C)** GSEA showing significant enrichment of gene sets involved in synapse organization and biogenesis and neuron differentiation among the genes upregulated by HOXC9. **(D)** Heatmap of select neuronal genes activated by HOXC9.

To gain a molecular understanding of HOXC9-induced differentiation, we conducted microarray gene expression profiling of human neuroblastoma BE(2)-C/Tet-Off/myc-HOXC9 cells, which express myc-tagged human HOXC9 and undergo neuronal differentiation in the absence of doxycycline [18] (Figure 1A). The profiling analysis identified a total of 2,370 genes that were differentially expressed (≥ +1.5 and ≤ −1.5 fold, *P* <0.01), with 879 genes being upregulated and 1,491 genes downregulated (Additional file 1: Table S1). Gene annotation enrichment analysis revealed that HOXC9-induced differentiation is characterized by a genome-wide coordination in transcriptional regulation of genes that control neuronal differentiation, cell cycle progression, and the DNA damage response.

### Global upregulation of neuronal genes

Gene Ontology (GO) analysis of the 879 HOXC9-upregulated genes by DAVID [29,30] revealed that they were significantly enriched for genes that control nervous system development such as neuron generation and differentiation, axonogenesis, and synapse formation and organization (Figure 1B and Additional file 2: Table S2, enrichment fold ≥ 2.0, false discovery rate (FDR) ≤1%). A total of 105 HOXC9-responsive genes were involved in nervous system development (Figure 1B), accounting for approximately 12% of the 879 genes upregulated by HOXC9. We obtained similar results with Gene Set Enrichment Analysis (GSEA), which showed significant enrichment of gene sets involved in synaptogenesis and neuron differentiation among the genes upregulated by HOXC9 (Figure 1C). Particularly significant was the activation of *ASCL1*, *GFRA3*, *RET*, and *NTN3* (Figure 1D). ASCL1, a member of the basic helix-loop-helix (bHLH) family of transcription factors, is a master regulator in the generation and differentiation of sympathetic neurons [31,32]. *GFRA3* encodes the glial cell line-derived neurotrophic factor (GDNF) family receptor alpha 3 (GFRα3), which forms a receptor complex with RET that preferentially binds the GDNF family ligand Artemin. This receptor signaling has a critical role in embryonic development of the sympathetic nervous system, promoting the survival, differentiation, axonal outgrowth, and target innervation of sympathetic neurons [33]. NTN3 (netrin 3) belongs to a family of extracellular proteins that promote axon growth and migration during the development of the nervous system [34]. Ingenuity Pathways Analysis (IPA) further revealed a network of HOXC9-upregulated genes relevant to the development and function of sympathetic neurons (Additional file 3: Figure S1). Together, these analyses demonstrate that HOXC9 activates a large number of neuronal genes, providing the molecular mechanism for its ability to induce neuronal differentiation of neuroblastoma cells.

### Global downregulation of cell cycle and DNA repair genes

**Figure 2:**
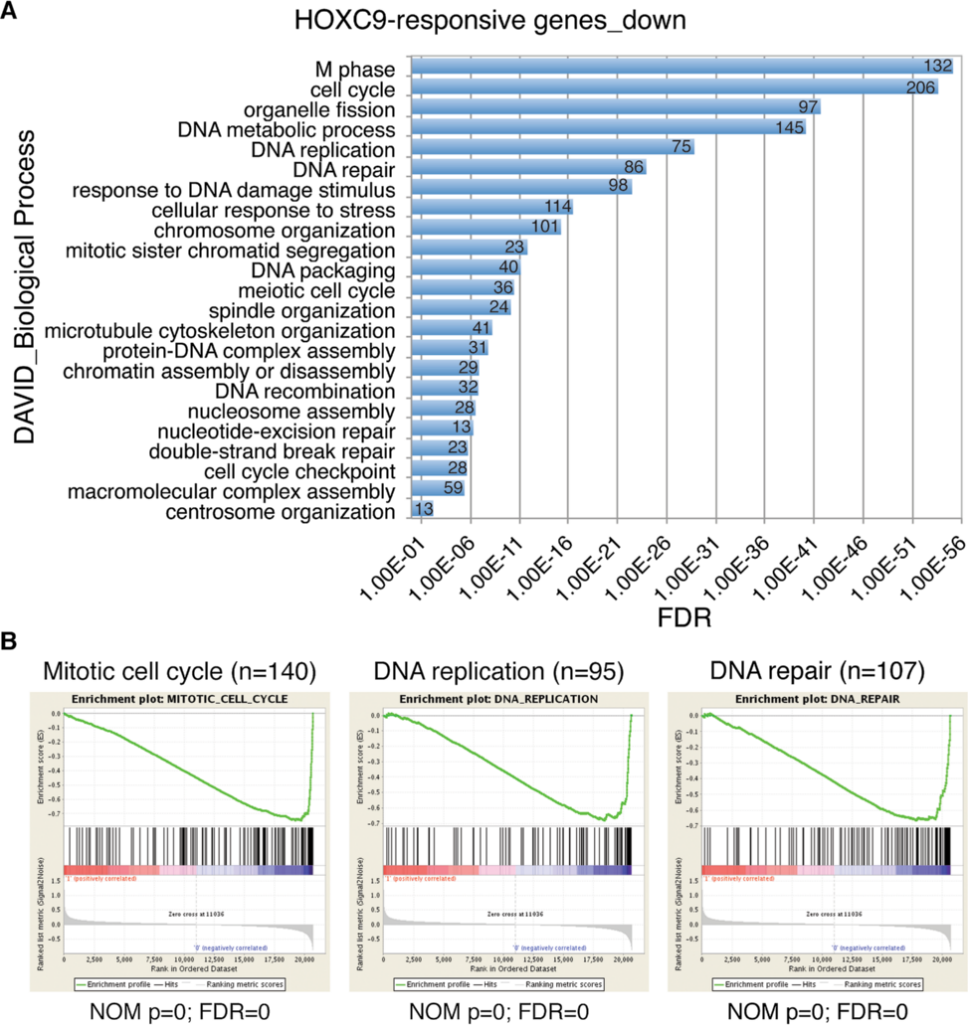
**Global downregulation of cell cycle and DNA repair genes by HOXC9. (A)** DAVID analysis of downregulated HOXC9-responsive genes for enriched GO biological process categories (enrichment fold > 2.0; FDR <1%). The number of genes for each biological process category is indicated. **(B)** GSEA showing significant enrichment of gene sets involved in mitotic cell cycle, DNA replication and DNA repair among the genes downregulated by HOXC9.

GO analysis of the 1,491 HOXC9-downregulated genes revealed that they were remarkably enriched for genes that control cell cycle progression and the DNA damage response (Figure 2A and Additional file 4: Table S3, enrichment fold ≥ 2.0; FDR ≤1%). The analysis identified 206 genes involved in cell cycle regulation and 98 genes in the DNA damage response (Figure 2A). Similarly, GSEA showed that among the genes downregulated by HOXC9, those regulating mitotic cell cycle, DNA replication and DNA repair were significantly enriched (Figure 2B). IPA further revealed that the downregulated genes include most of cyclin (CCN) and cyclin-dependent kinase (CDK) genes, and genes that control DNA replication, mitosis, double-strand break (DSB) repair, base excision repair (BER), nucleotide excision repair (NER), mismatch repair (MMR), and Fanconi anemia (FA)-mediated repair (Additional file 3: Figures S2A-S2E and Additional file 4: Table S3). These findings suggest that global downregulation of cell cycle and DNA repair genes is the primary cause of the cell cycle arrest and attenuation of the DNA damage response associated with neuronal differentiation.

### Genome-wide mapping of HOXC9-binding sites

**Figure 3:**
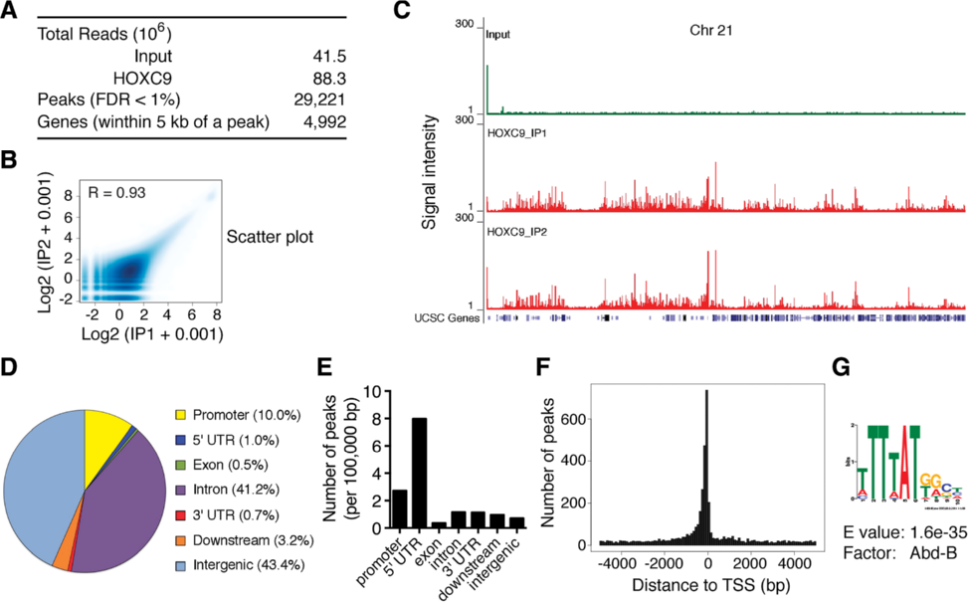
**ChIP-seq analysis of HOXC9 genomic distribution. (A)** Summary of HOXC9-binding peaks and genes identified by ChIP-seq. **(B-C)** Scatter plot analysis **(B)** and ChIP-seq tag profiles of chromosome 21 **(C)** showing a high correlation between two independent HOXC9 ChIP-seq samples. **(D)** Pie chart showing genomic distribution of HOXC9-binding peaks relative to RefSeq functional categories including promoters (within 5 kb upstream of TSS), 5’ UTRs, exons, introns, 3’ UTRs, downstream (within 5 kb downstream of the gene), and intergenic regions (outside −5 ~ +5 kb of genes). **(E)** Bar graph showing the distribution of HOXC9-binding peaks relative to the functional categories after normalization to the size of each category in 100 kb. **(F)** Histogram showing the distribution of HOXC9-binding peaks relative to the nearest TSS. Peaks were combined in 100 bp. **(G)** Web logo showing the top enriched motif present in HOXC9 ChIP-seq peaks, which corresponds to the binding site for Abd-B, the *Drosophila* ortholog of mammalian HOX9-13 paralogs.

We next asked how HOXC9 coordinates the expression of distinct sets of genes: the upregulation of genes critical for nervous system development and the downregulation of genes essential for cell cycle progression and the DNA damage response. Mechanistically, HOXC9 could function through a few master transcription factors, which in turn regulate their own subsets of target genes that work together to drive differentiation. Alternatively, HOXC9 could directly regulate distinct sets of genes to coordinate the cellular events associated with differentiation. To test these models, we conducted two independent anti-HOXC9 chromatin immunoprecipitation assays followed by massively parallel sequencing of the enriched DNA fragments (ChIP-seq) for genome-wide mapping of HOXC9-binding sites. We identified a total of 29,221 HOXC9-binding peaks with FDR less than 1% (Figure 3A and Additional file 5: Table S4). Scatter plot analysis (Figure 3B, R = 0.93, correlation coefficient) and ChIP-seq tag profiles (Figure 3C) demonstrated that the mapping data were highly reproducible between the two independent HOXC9 ChIP-seq samples. We next analyzed the distribution of HOXC9-binding peaks within the genome that was classified into functional categories including promoters (within 5 kb upstream of the transcription start site, TSS), 5’-untranslated regions (5’-UTRs), exons, introns, 3’-UTRs, downstream (within 5-kb downstream of the gene), and intergenic regions (outside −5 ~ +5 kb of genes). The analysis revealed that a majority of HOXC9-binding peaks were localized in introns (41.2%) and intergenic regions (43.4%) (Figure 3D). However, after normalization to the size of these functional regions, it became clear that HOXC9-binding peaks were highly enriched in gene promoters and 5’-UTRs (Figure 3E and F). Analysis of the sequences covered by HOXC9-binding peaks with the motif-finding program MEME revealed that the most enriched binding motif (T/ATTTAT, E value = 1.6e-35) corresponds to the *Drosophila* Abd-B motif (MA0165.1, Figure 3G) and is highly homologous to the mouse Hoxc9-binding motif (ATTTAT) [35]. HOXC9 is a mammalian ortholog of the *Drosophila* Hox protein Abd-B. Thus, myc-tagged HOXC9 binds to cognate sequences in human neuroblastoma cells.

### Genome-wide identification of HOXC9 target genes

The ChIP-seq assay revealed that a total of 4,992 genes contained at least one HOXC9-binding peak within 5-kb upstream or downstream of their genomic loci (Figure 3A and Additional file 6: Table S5). We next combined the anti-HOXC9 ChIP-seq data with the HOXC9 microarray data to generate a list of genes that were bound by HOXC9 and whose expression levels were significantly changed as a result of HOXC9 induction (≥ +1.5 and ≤ −1.5 fold, *P* <0.01). The analysis revealed that 954 genes or 40.3% of the 2,370 HOXC9-responsive genes are direct targets of HOXC9, with 445 and 509 genes being upregulated and downregulated, respectively (Additional file 7: Table S6). GO analysis of HOXC9 direct target genes revealed a transcriptional program characterized by coordinated regulation of genes critical for neuron differentiation, cell cycle progression, and the DNA damage response.

### HOXC9 directly induces a large number of neuronal genes

**Figure 4:**
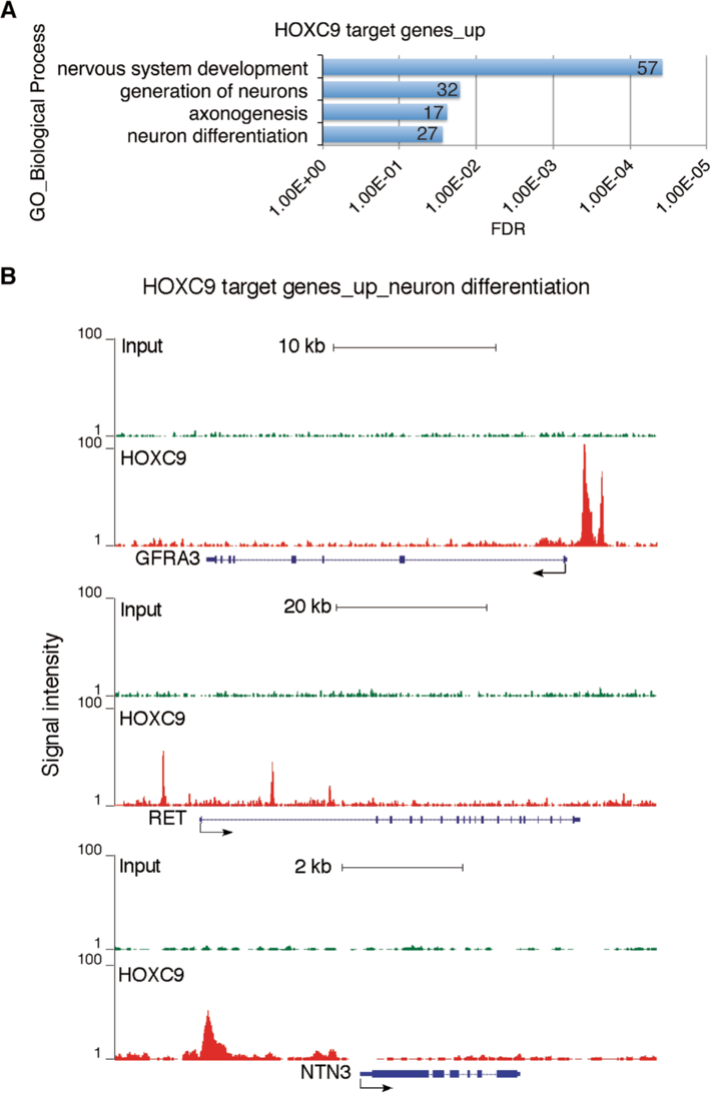
**HOXC9 directly induces a large number of neuronal genes. (A)** DAVID analysis of upregulated HOXC9 target genes for enriched GO biological process categories (enrichment fold > 2.0; FDR < 5%). The number of genes for each biological process category is indicated. **(B)** ChIP-seq tag profiles showing HOXC9 binding to representative upregulated HOXC9 target genes involved in nervous system development (*GFRA3*, top; *RET*, middle; *NTN3*, bottom).

The only sets of genes that were significantly enriched among the upregulated HOXC9 direct target genes are those exclusively involved in nervous system development, particularly the generation and differentiation of neurons and axonogenesis (Figure 4A and Additional file 8: Table S7, enrichment fold ≥ 2.0, FDR ≤ 5%). The 57 HOXC9 direct target genes account for 54.3% (57/105) of the HOXC9-responsive genes involved in nervous system development (Figure 1B). Among them are *ASCL1*, *GFRA3*, *RET*, and *NTN3*. Figure 4B shows the ChIP-seq tag profiles of HOXC9 binding to the promoter regions of *GFRA3*, *RET*, and *NTN3*. As discussed above, these genes have a critical role in sympathetic neurogenesis and axonogenesis.

### HOXC9 directly represses a large number of genes essential for cell cycle progression and the DNA damage response

**Figure 5:**
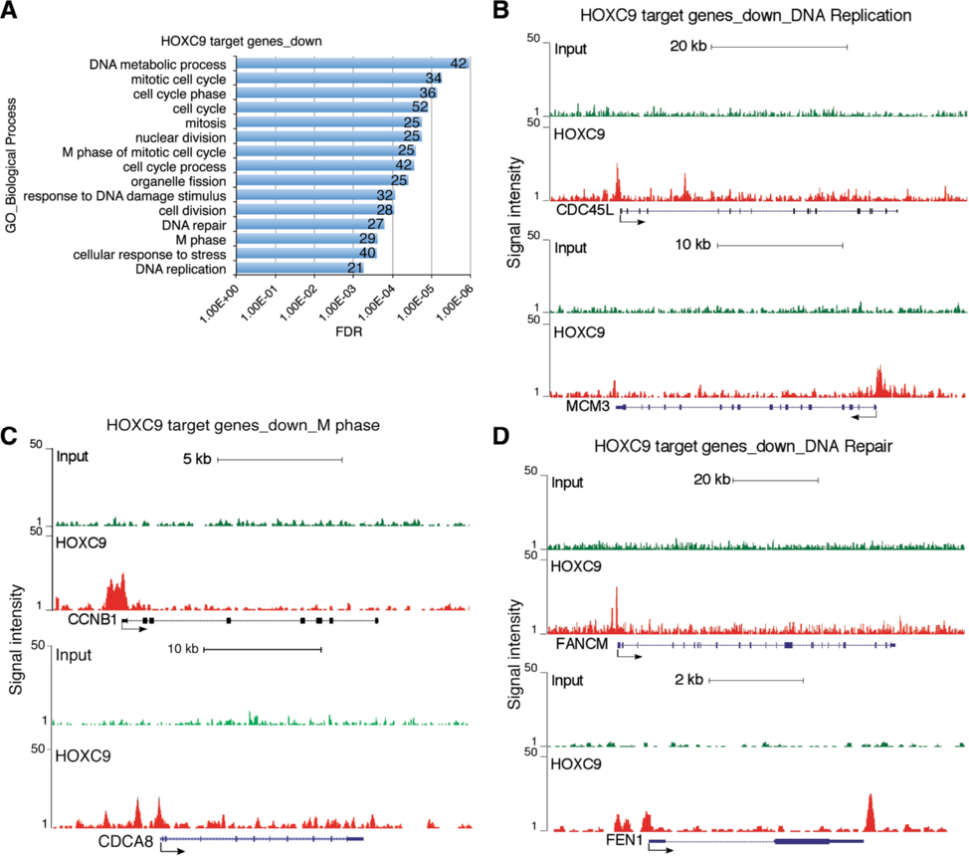
**HOXC9 directly represses a large number of cell cycle and DNA repair genes. (A)** DAVID analysis of downregulated HOXC9 target genes for enriched GO biological process categories (enrichment fold > 2.0; FDR <1%). The number of genes for each biological process category is indicated. **(B-D)** ChIP-seq tag profiles showing HOXC9 binding to representative downregulated HOXC9 target genes involved in DNA replication **(B)**, mitosis **(C)**, and DNA repair **(D)**.

GO analysis of the downregulated HOXC9 direct target genes revealed that they were significantly enriched for genes that control cell cycle progression and the DNA damage response (Figure 5A and Additional file 9: Table S8), enrichment fold ≥ 2.0, FDR ≤ 1%). The analysis identified 52 cell cycle genes that were directly repressed by HOXC9 (Figure 5A), accounting for 25.2% (52/206) of the HOXC9-responsive genes involved in cell cycle regulation (Figure 2A). It was particularly striking that the vast majority of the HOXC9-repressed cell cycle genes are involved in the control of the M phase (n = 25) and DNA replication (n = 21) (Figure 5A). Figure 5B and C show the association of HOXC9 with the promoter regions of representative cell cycle genes, including *CDC45L* and *MCM3* (DNA replication), and *CCNB1* and *CDCA8* (M phase). CDC45L and MCM3 are components of the replicative complex that catalyzes DNA replication during the S phase [36], while CDCA8, also known as BOREALIN, is a component of the chromosomal passenger complex essential for mitosis and cell division [37].

We also identified 32 genes associated with the DNA damage response that were directly repressed by HOXC9 (Figure 5A), accounting for 32.7% (32/98) of the HOXC9-responsive genes involved in the DNA damage response. Figure 5D shows the binding of HOXC9 to the promoter of *FANCM* and to both the promoter and 3’ region of *FEN1*. FANCM is a component of the FANCM–FAAP24–MHF protein complex that binds to DNA with interstrand cross-links and is responsible for recruiting the FA core complex to the damaged site [38]. FEN1 (flap endonuclease 1) is essential for DNA replication and repair by removing RNA and DNA 5' flaps [39].

Collectively, these findings suggest that HOXC9 directly regulates the expression of distinct sets of genes to coordinate the molecular and cellular processes characteristic of neuronal differentiation.

### HOXC9 targets E2F6 to the promoters of cell cycle genes

We next sought to determine the molecular basis for HOXC9 regulation of gene expression by identifying HOXC9-interacting proteins. We used a myc-tag antibody to isolate myc-HOXC9 and its associated proteins from nuclear extracts of BE(2)-C/Tet-Off/myc-HOXC9 cells cultured in the absence of doxycycline for 6 days (Additional file 3: Figure S3A). Mass spectrometric analysis of two independent samples identified E2F6 as a HOXC9-interacting protein (Additional file 3: Figure S3B), a well characterized transcriptional repressor that plays a major role in repressing E2F-responsive genes essential for cell proliferation [40]. It is known that E2F family proteins (E2F1-6) share the same core consensus G/CTTTG/C binding site [41]. Interestingly, GSEA revealed significant enrichment of the E2F-binding motif among the genes downregulated by HOXC9 (Additional file 3: Figure S3C). Taken together, these observations suggest that E2F6 has an important role in HOXC9-mediated repression of cell cycle genes.

**Figure 6:**
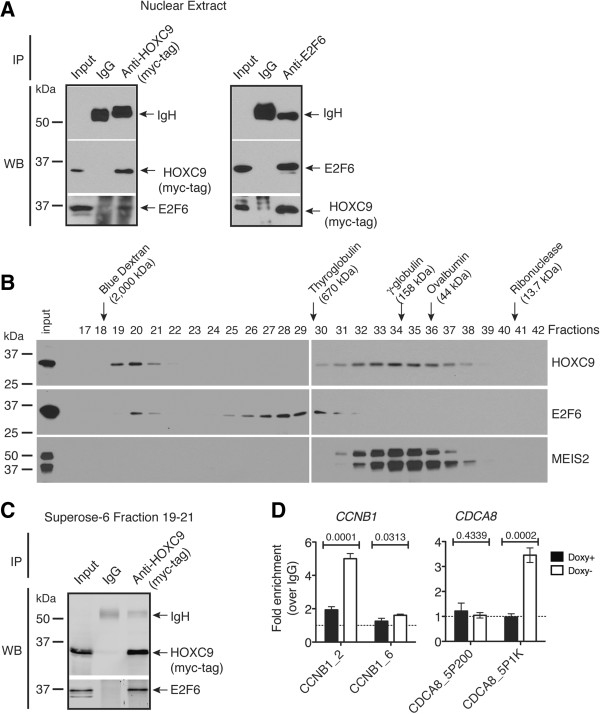
**HOXC9 interacts with E2F6 and recruits it to cell cycle genes. (A)** Reciprocal Co-IP of myc-HOXC9 and E2F6 in nuclear extracts from BE(2)-C/Tet-Off/myc-HOXC9 cells cultured in the absence of doxycycline (Doxy) for 6 days. **(B)** Size-exclusion chromatography analysis of complexes containing myc-HOXC9, E2F6 or MEIS2 in nuclear extracts of BE(2)-C/Tet-Off/myc-HOXC9 cells cultured in the absence of Doxy for 6 days. **(C)** Co-IP of myc-HOXC9 and E2F6 in pooled Superose-6 fractions 19–21. **(D)** ChIP-qPCR analysis showing E2F6 binding to specific promoter regions of the cell cycle genes *CCNB1* and *CDCA8* in BE(2)-C/Tet-Off/myc-HOXC9 cells before (Doxy+) and after (Doxy-) HOXC9 induction. Dashed lines indicate IgG control. Error bars represent SD (n = 3). Data were analyzed with unpaired, two-tailed Student’s *t*-test and *p* values are indicated.

To corroborate the finding of mass spectrometry, we performed co-immunoprecipitation (Co-IP) experiments using nuclear extracts from BE(2)-C/Tet-Off/myc-HOXC9 cells cultured in the absence of doxycycline for 6 days. The myc-tag antibody, but not control IgG, precipitated myc-HOXC9 and E2F6 (Figure 6A, left panel). Reciprocally, an E2F6 antibody precipitated E2F6 and myc-HOXC9 (Figure 6A, right panel). We next performed size-exclusion chromatography using the same nuclear extracts. Immunoblot analysis revealed the presence of HOXC9 (~31 kDa) in two complexes: the larger complex (peak at fraction 20) had an estimated molecular mass of ~1,800 kDa and the other (peak at fraction 34) of ~250 kDa (Figure 6B). A significant amount of endogenous E2F6 (~36 kDa) co-eluted with the 1,800-kDa HOXC9 complex, whereas MEIS2 (~37-49 kDa), which interacts with HOX proteins and functions as a HOX cofactor [12], exclusively co-eluted with the 250 kDa-HOXC9 complex (Figure 6B). Co-IP experiments using pooled fractions confirmed the association of HOXC9 with E2F6 within the larger complex (Figure 6C).

To determine whether the HOXC9-E2F6 interaction plays a role in recruiting E2F6 to HOXC9 target genes in vivo, we performed anti-E2F6 ChIP using BE(2)-C/Tet-Off/myc-HOXC9 cells before and after HOXC9 induction. HOXC9 induction had no apparent effect on E2F6 expression as determined by microarray gene expression profiling (−1.003 fold). ChIP-qPCR assay revealed that E2F6 was recruited to specific promoter regions of the cell cycle genes *CCNB1* and *CDCA8* only after HOXC9 induction (Figure 6D). By contrast, no significant binding of E2F6 to the *NEFM* promoter was observed before and after HOXC9 induction (Additional file 3: Figure S4A). As reported previously, *NEFM* is a neuronal gene directly activated by HOXC9 during differentiation [18] (See also Additional file 3: Figure S4B). Together, these data suggest that elevated levels of HOXC9 facilitate the formation of a repressive complex with E2F6, which is then recruited to cell cycle but not neuronal genes during differentiation.

### E2F6 is essential for HOXC9-induced cell cycle arrest and transcriptional repression of cell cycle genes

**Figure 7:**
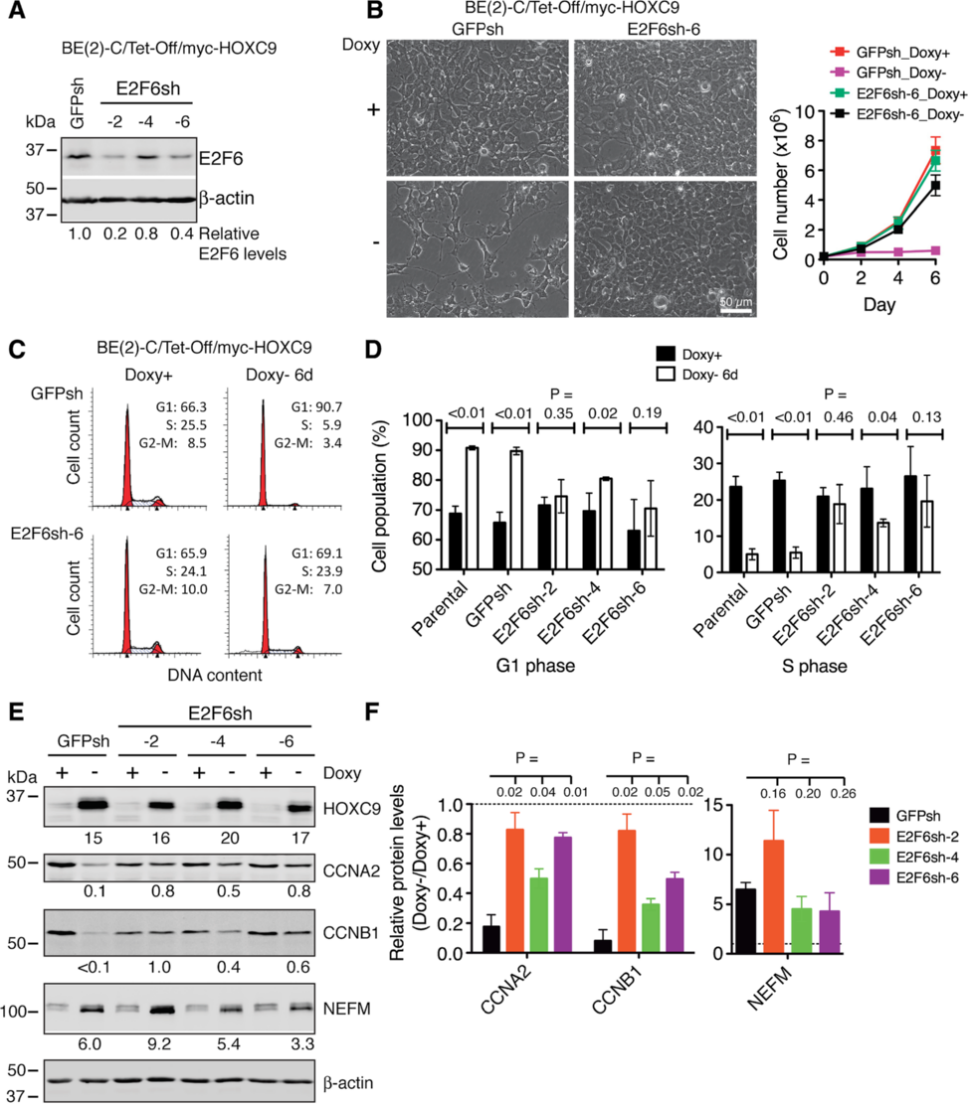
**E2F6 is essential for HOXC9 induction of G1 arrest and repression of cell cycle genes. (A)** Immunoblot analysis of E2F6 levels in BE(2)-C/Tet-Off/myc-HOXC9 cells infected with lentiviruses expressing shRNA against *GFP* or various coding regions of *E2F6*. E2F6 levels were quantified against β-actin. **(B-D)** Phase contrast imaging and growth assay **(B)** and cell cycle analysis **(C, D)** showing E2F6 knockdown abrogated HOXC9-induced growth arrest. Error bars, SD (n = 4). **(E-F)** Immunoblot analysis **(E)** and quantification **(F)** showing that E2F6 knockdown abrogated HOXC9 repression of cyclins, but not HOXC9 induction of NEFM. HOXC9, CCNA2, CCNB1 and NEFM levels were quantified against β-actin with the protein levels in GFPsh-expressing cells cultured in the presence of doxycycline (Doxy+) were defined as 1.0 (dashed lines). Error bars, SD (n = 3). Data in **(D)** and **(F)** were analyzed with unpaired, two-tailed Student’s *t*-test and *p* values are indicated.

To determine the functional significance of the HOXC9-E2F6 interaction, we examined the effect of E2F6 knockdown on HOXC9-induced growth arrest. We depleted E2F6 using short hairpin RNA (shRNA) sequences targeting different coding regions of the human *E2F6* gene (Figure 7A). Cells with E2F6 knockdown were highly resistant to HOXC9-induced G1 arrest, showing continued cell proliferation (Figure 7B) and cell cycle progression (Figure 7C) following HOXC9 induction. This was accompanied by a marked decrease in the population of cells in the G1 phase and a significant increase in the population of cells in the S phase (Figure 7D). In addition, E2F6 knockdown largely abrogated the ability of HOXC9 to repress cyclin A2 and B1 expression, but had no significant effect on HOXC9 induction of NEFM (Figure 7E and F), a finding consistent with the observation of no significant E2F6 binding to the *NEFM* promoter during HOXC9-induced differentiation (Additional file 3: Figure S4A). Together, these findings identify an essential and specific role for E2F6 in HOXC9 induction of growth arrest and repression of cell cycle genes.

## Discussion

**Figure 8:**
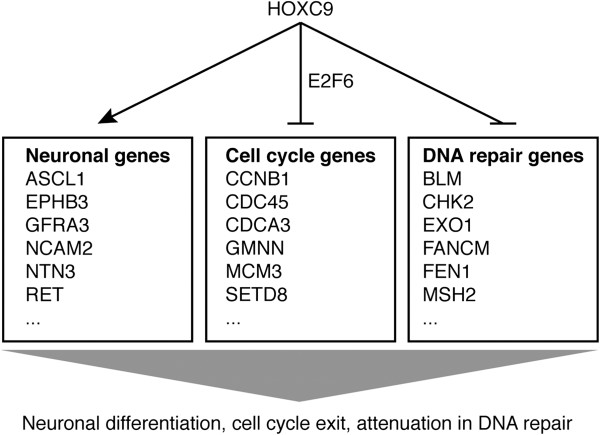
HOXC9 directly regulates the expression of distinct sets of genes to coordinate diverse cellular events associated with neuronal differentiation.

In this report, we present evidence for a master regulator of development with the capacity to coordinate diverse cellular events characteristic of neuronal differentiation by simultaneously and directly regulating distinct sets of genes (Figure 8). Through gene expression profiling, we show that HOXC9-induced neuronal differentiation is characterized at the molecular level by transcriptional regulation of 2,370 genes, with global activation of genes that promote nervous system development and repression of genes that are essential for cell cycle progression and the DNA damage response. Moreover, through a combination of genome-wide mapping of HOXC9 binding sites and gene expression profiling, we show that HOXC9 directly regulates the expression of 954 genes, ~40% of the 2,370 HOXC9-responsive genes, including a large number of genes required for neuronal differentiation, cell cycle progression and the DNA damage response. Finally, we identify an essential role for E2F6 in HOXC9 repression of cell cycle genes and induction of G1 arrest.

Our findings that HOXC9 can both activate and repress gene transcription are consistent with previous observations from the study of spinal cord development in chick and mouse embryos. In the developing spinal cord, Hoxc9 functions as a transcription activator to promote the fate of preganglionic motor column (PGC) neurons [42], most likely through its interaction with the transcription factor FoxP1 [43,44]. However, Hoxc9 can also specify the fate of hypaxial motor column (HMC) neurons by repressing the *Hox* genes that promote the switch of HMC neurons to the lateral motor column (LMC) neurons [35]. Importantly, our study further demonstrated that within the same population of neuroblastoma cells, HOXC9 could simultaneously activate the genes that promote neuronal differentiation and repress the genes that are essential for cell cycle progression and the DNA damage response. While the molecular basis for the transcription activator function of HOXC9 in neuroblastoma cells remain to be defined, we showed that the ability of HOXC9 to repress cell cycle genes depended on its interaction with the transcription repressor E2F6, a member of the E2F family of transcription factors that have a critical role in the control of cell proliferation [40].

Cellular differentiation is tightly linked to cell cycle exit, with the differentiated cell containing the G1 content of DNA. The molecular mechanism that couples cell cycle exit and differentiation is not well understood, although it is generally recognized that cell cycle regulators influence differentiation, and cell fate determinants influence the cell cycle [45-48]. A primary example is the CDK inhibitor p27^Kip1^ as a key regulator that links cell cycle exit and differentiation during development. p27^Kip1^ induces G1 arrest by associating with CDK/cyclin complexes and inhibits their kinase activity [49]. Overexpression of p27^Xic1^, a *Xenopus* homolog of p27^Kip1^, in *Xenopus* retina glial progenitor cells promotes both cell cycle exit and differentiation [50]. Knockout and overexpression studies also demonstrate an important role of p27^Kip1^ in neuronal differentiation in the mouse cerebral cortex by stabilizing Neurogenin 2 [51], a proneural bHLH transcription factor with a central role in cortical neurogenesis [52]. On the other hand, cell fate determinants can also modulate the expression of p27^Kip1^ for coordinated regulation of cell cycle exit and differentiation. For instance, *Drosophila* proneural bHLH proteins cooperate with epidermal growth factor signaling to directly activate the transcription of *Dapaco*, a homolog of p21^Cip^/p27^Kip1^, during the differentiation of photoreceptor cells [53].

Our findings suggest an alternative mechanism for coupling cell cycle exit and differentiation. HOXC9 does not regulate the expression of CDK inhibitors, including p27^Kip1^ and p21^Cip^, and overexpression of either p27^Kip1^ or p21^Cip^ fails to stop the proliferation of BE(2)-C cells [18]. Rather, HOXC9 induces G1 arrest by directly repressing a large number of genes essential for cell cycle progression through the S to M phases, including cyclin B1, CDCA3, CDCA8, BUB1B, MCM3 and MCM8. This transcriptional repression function of HOXC9 requires E2F6. We found that HOXC9 interacts with E2F6 and recruits it specifically to the promoters of cell cycle genes. E2F6 lacks a transactivation domain and functions as a transcriptional repressor for E2F-responsive genes that drive cell proliferation [54-58]. Mechanistically, E2F6 interacts with chromatin modifiers with transcription repressor activity to establish a repressive chromatin structure. These chromatin modifiers include the DNA methyltransferase Dnmt3b [59] and polycomb-group (PcG) proteins [60-63]. In our study, we identified HOXC9 and E2F6 within a complex of approximately 1,800 kDa. Whether this complex contains chromatin modifiers is currently under investigation.

Terminal cell differentiation is also tightly associated with a global reduction in DNA damage repair activities [1-3]. The underlying molecular mechanism is not well understood. It has been reported that E1 ubiquitin-activating enzyme can complement nucleotide-excision repair deficiency in extracts from differentiated macrophages, suggesting a role of ubiquitination in the control of the DNA damage response during differentiation [64]. Our study revealed that in HOXC9-induced neuronal differentiation, attenuation of the DNA damage response resulted from global transcriptional repression of DNA repair genes. This finding provides a molecular mechanism for the long observed differentiation-induced radiosensitivity in neuroblastoma cells [20,24-27]. For HOXC9-induced differentiation, a total of 98 genes with functions in the DNA damage response were significantly downregulated. These genes are involved in all types of DNA damage checkpoints and repair pathways. Importantly, we show that 32 of the 98 genes are direct targets of HOXC9. Thus, to a large extent, HOXC9 coordinates neuronal differentiation and attenuation of DNA repair activities by simultaneously activating neuronal genes and repressing DNA repair genes. Since the DNA damage response and DNA replication machineries share many components, we speculate that the downregulation of DNA repair genes during differentiation is a consequence of repression of cell cycle genes, particularly those involved in DNA replication.

The stem cell model of cancer attributes cancer growth to a subpopulation of cancer stem cells. It has been shown recently that cancer stem cells are intrinsically resistant to ionizing radiation and chemotherapy, as a result of enhanced checkpoint activation and more effective DNA damage repair [7-10]. Since differentiation is associated with global downregulation of DNA repair activities, a combination of differentiation-inducing agents and irradiation or chemotherapy may prove to be a more effective therapeutic strategy for targeting cancer stem cells.

## Conclusions

Using neuroblastoma cell differentiation as an experimental system, we delineate a molecular mechanism by which HOXC9 coordinates diverse cellular processes associated with differentiation by directly activating and repressing the transcription of distinct sets of genes.

## Methods

### Cell culture and growth assays

The human neuroblastoma cell line BE(2)-C (CRL-2268, ATCC) with Tet-Off inducible expression of myc-tagged human HOXC9 has been described previously [18]. For E2F6 knockdown, BE(2)-C/Tet-Off/myc-HOXC9 cells were infected with lentiviruses expressing shRNA against E2F6 (TRCN013819, E2F6sh-2, TTTCGAGTTAAATAAACCAGC; TRCN013821, E2F6sh-4, ATTGGTGATGTCATACACTCT; TRCN018201, E2F6sh-6, ATCCAAAGCATCTTCCATTGC; Thermo Fisher Scientific). Cells were cultured in a 1:1 mixture of DMEM and Ham’s nutrient mixture F12 supplemented with 10% fetal bovine serum (Invitrogen-Gibco) in the presence or absence of doxycycline. Cells were examined and phase contrast images captured using an Axio Observer microscope and AxioVision software (Carl Zeiss MicroImaging), and viable cell numbers were determined by trypan blue exclusion assay. For cell cycle analysis, cells were fixed in 70% ethanol, incubated with ribonuclease A (Sigma-Aldrich), and stained with 20 μg/ml propidium iodide (Invitrogen-Gibco). Samples were analyzed using a FACSCalibur system and ModFitLT V3.2.1 software (BD Bioscience).

### Microarray gene expression profiling

Total RNA was isolated using Trizol (Invitrogen) from three independent samples of BE(2)-C/Tet-Off/myc-HOXC9 cells cultured in the presence or absence of doxycycline for 6 days. RNA was measured and quality assessed by a NanoDrop spectrophotometer and an Agilent 2100 Bioanalyzer (Agilent Technologies). Affymetrix microarray analysis was performed using the Human Gene 1.0 ST microarray chip. Data were normalized, significance determined by ANOVA, and fold change calculated with the Partek Genomics Suite (Partek Inc.). Gene annotation enrichment analysis was performed with DAVID v6.7 [30], GSEA [65], and IPA (Ingenuity® Systems http://www.ingenuity.com) for all significantly changed genes (≥ +1.5 and ≤ −1.5 fold, *P* < 0.01).

### ChIP-seq and ChIP-qPCR

Two independent preparations of BE(2)-C/Tet-Off/myc-HOXC9 cells cultured in the presence or absence of doxycycline for 6 days were used for ChIP. Cross-linked chromatin DNA was sheared through sonication and immunoprecipitated using mouse anti-myc tag (clone 4A6, Millipore) or mouse anti-E2F6 (sc-53273, Santa Cruz Biotechnologies) according to the published procedure [66]. For ChIP-seq, libraries were generated from ChIP genomic DNA samples according to the Illumina ChIP-seq library construction procedure, and sequenced using Illumina Genome Analyzer IIx with a read length of 36 or 76 bp. For ChIP-qPCR, ChIP genomic DNA samples were assayed in triplicate by PCR using an iQ5 real-time PCR system (Bio-Rad) and the following primer sets that cover the promoter regions of *CCNB1* (CCNB1_2 and CCNB1_6), *CDCA8* (CDCA8_5P200 and CDCA8_5P1K), and *NEFM* (NEFM_5P1 and NEFM_5P2): CCNB1_2: CCAGAGAGTTGTTGCAACGAT, CTGGAGAGCAGTGAAGCCAGT; CCNB1_6: GGAAGGATTGATCAAACCCAG, AGTCACGGATCCGAAAGAAGG; CDCA8_5P200: GGTATTGCAGAGCCGCCA, CCTCCCCACCAACCCACC; CDCA8_5P1K: TGGTGCCCATCAGGAGCC, GGCTATGGGAGTGATAATC; NEFM_5P1: GCAGAAAGTAATAAGCAACAA, CCTGCCTTCTGTAAAGTATTG; NEFM_5P2: CCTTTCCTGATTACTTACTGA, AGGGACTCCAGACCGAAATAG.

### ChIP-seq data analysis

Raw Illumina sequencing reads from the two independent ChIP replicates (rep1, GEO GSM848788 and rep2, GEO GSM848789) in the FASTQ format were cleaned using in-house scripts by trimming sequencing adaptors and low quality bases in both ends (Q < 67 in Illumina 1.5). Cleaned sequences were then mapped to the human genome (hg19) using Novoalign v2.07 for identifying the reads that were mapped uniquely to a single genomic locus. The identified reads from the rep1 ChIP sample (GEO GSM848788) were used for peak calling with Model-based Analysis of ChIP-Seq (MACS v1.4) [67], and only those peaks with FDR <1% were compared with RefSeq genes in the UCSC genome browser and classified into functional categories such as promoters, 5’-UTRs, exons, introns, 3’-UTRs, downstream, and intergenic regions. To measure the correlation of two HOXC9 replicates, we used 200 bp non-overlapping windows where a tag density is defined as the number of reads in a window. We calculated Pearson correlation coefficient with R > 0.9 being highly correlated. For motif analysis, we extracted 100 bp flanking sequences from predicted peak summits and ran MEME for identifying statistically overrepresented motifs. We performed MAST to search motifs in the peaks using the model built by MEME.

### Identification of HOXC9 target genes

Genes with HOXC9-binding peaks that are non-intergenic (i.e., within −5 ~ +5 kb of genes) were defined as HOXC9 target genes. To correlate HOXC9 binding to gene expression, we combined the HOXC9 ChIP-seq data with the HOXC9 microarray data using in-house scripts to generate a list of the genes whose regulatory elements are bound by HOXC9 and whose expression levels are significantly changed (≥ +1.5 and ≤ −1.5 fold, *P* < 0.01) as the result of HOXC9 induction. The significantly up- and down-regulated HOXC9 target genes were then subjected to gene annotation enrichment analysis with DAVID v6.7, GSEA, and IPA.

### Immunoprecipitation and mass spectrometric analyses

BE(2)-C/Tet-Off/myc-HOXC9 cells were cultured in the absence of doxycycline for 6 days and nuclear extracts were prepared following the Dignam protocol [68] except that buffer C contained 300 mM NaCl. Extracts from 1 × 10^7^ cells were incubated with Protein A/G beads (Invitrogen) coated with 4 μg mouse anti-Myc tag (clone 4A6, Millipore) or mouse IgG for overnight at 4°C. The beads were washed 3 times with buffer C containing 150 mM NaCl, dried in a SpeedVac, re-suspended in a buffer containing 8M urea, 5 mM DTT and 100 mM ammonium bicarbonate, and alkylated with 15 mM iodoacetamide for 1 hour. After alkylation, unreacted iodoacetamide was removed by 15 mM DTT and the urea concentration was diluted to ~1M with a buffer containing 50 mM ammonium bicarbonate and 2 mM CaCl_2_. Immunoprecipitated proteins were digested with 14 ng/μl sequencing grade trypsin (Promega) for 24 hours at 37°C. The digests were desalted with a Micro Trap desalting cartridge (Michrom BioResources), and tryptic peptides eluted with LC-MS Solvent B (90/10/0.05%: Acetonitrile/water/heptafluorobutyric acid) and dried in a SpeedVac. The digests were analyzed by Nano-HPLC using a Nano Trap column (CL5/61241/00, Michrom BioResources) and an Agilent 1200 Series Nano pump (Agilent Technologies) equipped with a refrigerated autosampler. An Agilent 1200 Series Capillary LC loading pump was used to introduce the sample onto a Captrap cartridge for sample concentration and de-salting.

Data-dependent MS and MS/MS spectra were acquired on an LTQ Orbitrap Discovery (Thermo Fisher Scientific) using 2 micro-scans, with a maximum injection time of 200 ms with 2 Da peak isolation width. Six scan events were recorded for each data acquisition cycle. The first scan event, acquired by the FTMS, was used for full scan MS acquisition from 300–2000 *m*/*z*. Data were recorded in the Centroid mode only. The remaining five scan events were used for collisionally activated dissociation (CAD): the five most abundant ions in each peptide MS were selected and fragmented to produce product-ion mass spectra.

### Database searching and protein identification

All MS/MS data were analyzed using BioWorks Rev.3.3.1 SP1 (Thermo Fisher Scientific) and X!Tandem (thegpm.org). SEQUEST was set up to search NCBInr_Homosapiens_05262011.fasta (221863 entries) and the human.protein_RefSeq_01192012 database (33376), and X!Tandem was set up to search subsets of the databases. SEQUEST and X!Tandem were searched with a fragment ion mass tolerance of 0.80 Da and a parent ion tolerance of 10.0 PPM. Scaffold (Proteome Software) was used to validate MS/MS-based peptide and protein identifications. Peptide identifications were accepted if they could be established at greater than 95.0% probability as specified by the Peptide Prophet algorithm [69]. Protein identifications were accepted if they could be established at greater than 90.0% probability and contained at least 1 identified peptide. Protein probabilities were assigned by the Protein Prophet algorithm [70]. Proteins that contained similar peptides and could not be differentiated based on MS/MS analysis alone were grouped to satisfy the principles of parsimony. Single-peptide protein identification was accepted only if the protein was independently identified by both SEQUEST and X!Tandem.

### Size-exclusion chromatography

Size-exclusion chromatography was performed with a Superose-6 10/300 GL column (24 ml bed volume) and an AKTA purifier (GE Healthcare). Nuclear extracts (0.5 ml) were loaded onto the column equilibrated with PBS, and 0.5 ml fractions were collected and analyzed.

### Co-immunoprecipitation

Nuclear extracts or pooled Sepharose-6 fractions were incubated with protein A/G beads coated with mouse anti-Myc tag (clone 4A6), mouse anti-E2F6, or control mouse IgG for 2 hours at 4°C. After washing 3 times with PBS, the beads were suspended in standard SDS sample buffer and analyzed by immunoblotting.

### Immunoblotting

Unless indicated, all antibodies were from Santa Cruz Biotechnologies. Samples were suspended in SDS sample buffer and boiled. Proteins were separated on SDS-polyacrylamide gels, transferred to nitrocellulose membranes, and probed with the following primary antibodies: rabbit anti-cyclin A2 (sc-751, 1:200), rabbit anti-cyclin B1 (sc-752, 1:200), mouse anti-myc-tag (9E10, hybridoma supernatant, 1:10), rabbit anti-E2F6 (sc-22823, 1:200), mouse anti-MEIS2 (63-T, sc-81986, 1:400), mouse anti-NEFM (NF-09, sc-51683, 1:200), and rabbit anti-β-actin (600-401-886, Rockland Immunochemicals, 1:2000). Horseradish peroxidase-conjugated goat anti-mouse and goat anti-rabbit IgG were used as secondary antibodies. Proteins were visualized using a SuperSignal West Pico chemiluminescence kit (Pierce, Thermo Fisher Scientific) and quantified with ImageJ (National Institutes of Health). For visualization and quantification with the Odyssey system, goat anti-mouse IRDye 800, anti-rabbit IRDye 800, anti-mouse IRDye 680, and anti-rabbit IRDye 680 were used as secondary antibodies (LI-COR Biosciences).

### Statistics

All quantitative data were analyzed and presented with GraphPad Prism 5.0f for Mac using unpaired, two-tailed Student’s *t*-test.

## Abbreviations

BER: Base excision repair; CCN: Cyclin; CDK: Cyclin-dependent kinase; ChIP-seq: Chromatin immunoprecipitation and sequencing; Co-IP: Co-immunoprecipitation; DAVID: Database for annotation visualization and integrated discovery; DMEM: Dulbecco's modified eagle medium; DSB: Double-strand break; FA: Fanconi anemia; FDR: False discovery rate; FoxP1: Forkhead box protein P1; GSEA: Gene set enrichment analysis; HMC: Hypaxial motor column; IPA: Ingenuity pathways analysis; LC-MS: Liquid chromatography; Mass: Spectrometry; LMC: lateral motor column; MMR: Mismatch repair; NEFM: Neurofilament medium; NER: Nucleotide excision repair; PBS: Phosphate buffered saline; PGC: Preganglionic motor column; RA: Retinoic acid; TSS: Transcription start site; UTR: Untranslated region.

## Competing interests

The authors declare that they have no competing interests.

## Authors’ contributions

XW, JD, LY and H-FD performed experiments with the assistance of HC and LM in establishing and characterizing inducible HOXC9 expression cells, LM in microarray sample preparation, BJ and MR in ChIP, EJL in ChIP-seq library preparation, and LCN in mass spectrometry. J-HC YZ, and H-FD performed microarray and ChIP-seq data analyses. XW, JD, HC, and H-FD designed the study with the assistance of HS, JC, and SH H-FD wrote the manuscript with contributions from JD, J-HC, LCN and EJL. All authors read and approved the final manuscript.

## Supplementary Material

Additional file 1: Table S1 HOXC9-responsivel genes. Click here for file

Additional file 2: Table S2 GO analysis of upregulated HOXC9-responsive genes. Click here for file

Additional file 3 Supplemental Figures S1-S4. Click here for file

Additional file 4: Table S3 GO analysis of downregulated HOXC9-responsive genes. Click here for file

Additional file 5: Table S4 HOXC9-binding peaks. Click here for file

Additional file 6: Table S5 HOXC9-binding genes. Click here for file

Additional file 7: Table S6 HOXC9-target genes. Click here for file

Additional file 8: Table S7 GO analysis of upregulated HOXC9 direct target genes. Click here for file

Additional file 9: Table S8 GO analysis of downregulated HOXC9 direct target genes. The microarray and ChIP-seq data have been deposited in the NCBI Gene Expression Omnibus (GEO) with the accession number GSE34422 (http://0-www.ncbi.nlm.nih.gov.elis.tmu.edu.tw/geo/query/acc.cgi?acc=GSE34422). Click here for file
