# Supplementary material for: HOXC9 directly regulates distinct sets of genes to coordinate diverse cellular processes during neuronal differentiation
Source: BMC Genomics. 2013 Nov 25;14:830. doi: 10.1186/1471-2164-14-830 (PMC3906982; doi:10.1186/1471-2164-14-830)
Supplement: Additional file 3 — Supplemental Figures S1-S4. [file 1471-2164-14-830-S3.pdf]

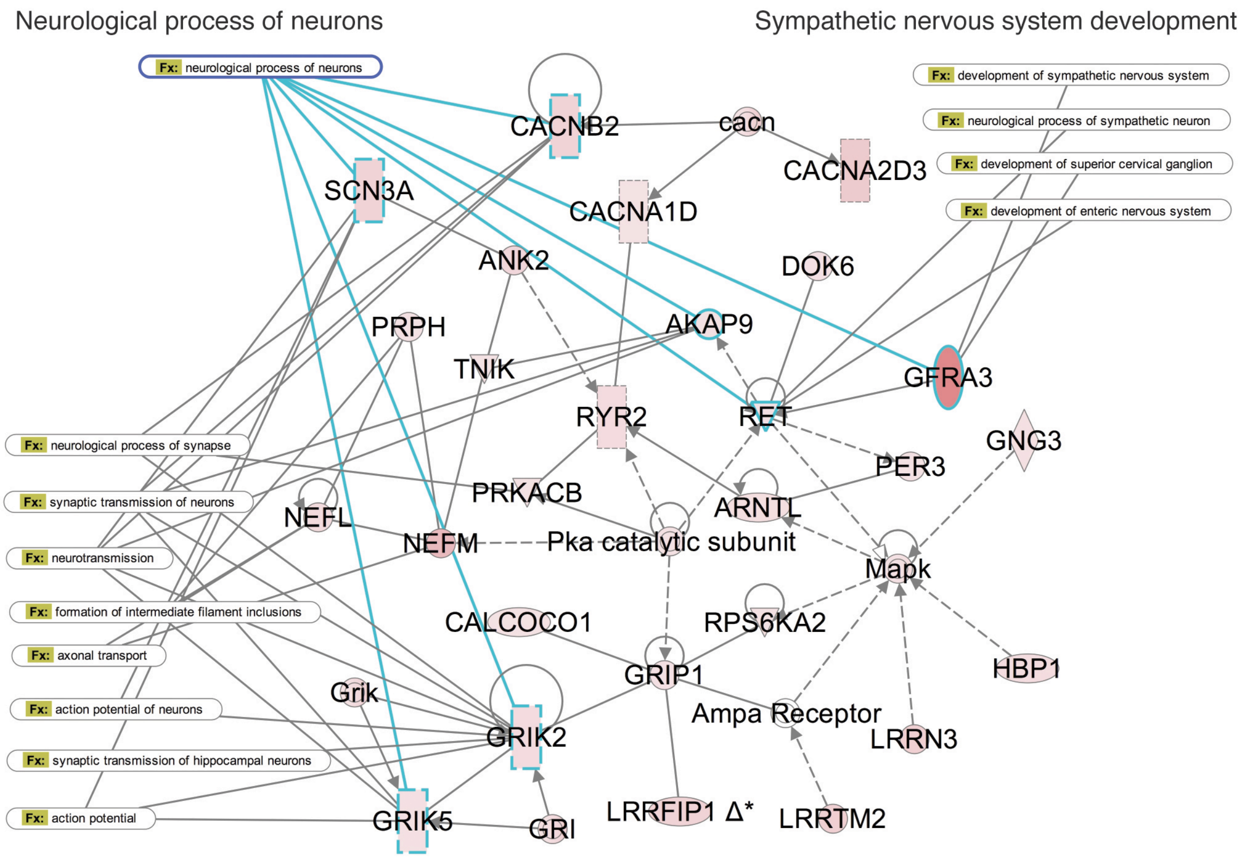

Figure S1 Global upregulation of neuronal genes by HOXC9. IPA of HOXC9-responsive neuronal genes, showing marked upregulation of genes important for the neurological process of neurons and the development of the sympathetic nervous system. Red color indicates a significant increase in expression with the color intensity being proportional to fold change. Solid lines indicate a direct relationship and dashed lines indicate an indirect interaction.

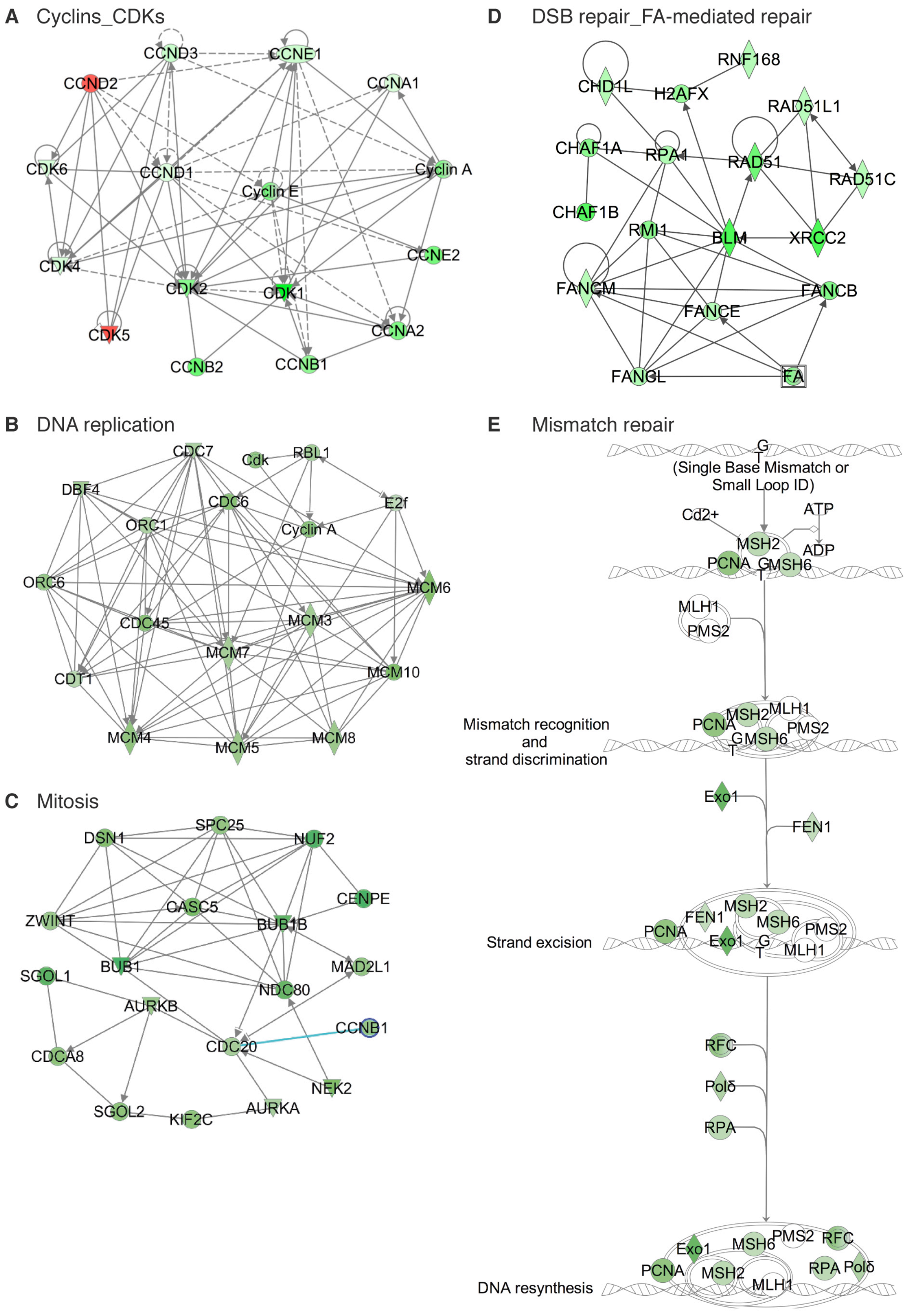
A Cyclins_CDKs
CCND3
CCNE1
CCND2
CCNA1
CDK6
CCND1
Cyclin A
Cyclin E
CDK4
CCNE2
CDK2
CDK1
CDK5
CCNA2
CCNB2
CCNB1
B DNA replication
CDC7
Cdk
RBL1
DBF4
CDC6
E2f
ORC1
Cyclin A
ORC6
MCM6
CDC45
MCM3
MCM7
CDT1
MCM10
MCM4
MCM5
MCM8
C Mitosis
DSN1
SPC25
NUF2
CENPE
ZWINT
CASC5
BUB1B
BUB1
MAD2L1
SGOL1
AURKB
NDC80
CCNB1
CDCA8
CDC20
NEK2
SGOL2
KIF2C
AURKA
D DSB repair_FA-mediated repair
RNF168
CHD1L
H2AFX
RAD51L1
CHAF1A
RPA1
RAD51
RAD51C
CHAF1B
RMI1
BLM
XRCC2
FANCM
FANCB
FANCE
FANCL
FA
E Mismatch repair
G
T
(Single Base Mismatch or
Small Loop ID)
Cd2+
ATP
MSH2
ADP
PCNA
G
MSH6
T
MLH1
PMS2
Mismatch recognition
and
strand discrimination
PCNA
MSH2
MLH1
PMS2
G
MSH6
T
Exo1
FEN1
Strand excision
PCNA
FEN1
MSH2
MSH6
PMS2
Exo1
G
T
MLH1
RFC
Polδ
RPA
DNA resynthesis
Exo1
MSH6
PMS2
RFC
PCNA
MSH2
MLH1
RPA
Polδ

Figure S2 Global downregulation of cell cycle and DNA damage response genes by HOXC9. IPA of HOXC9-responsive genes, showing downregulation of cyclins and CDKs (**A**), and genes essential for DNA replication (**B**), mitosis (**C**), double-strand break (DSB) repair and Fanconi anemia (FA)-mediated repair (**D**), and mismatch repair (**E**). Red and green colors indicate a significant increase and decrease in expression, respectively, with the color intensity being proportional to fold change. Solid and dashed lines indicate direct and indirect interactions, respectively.

A

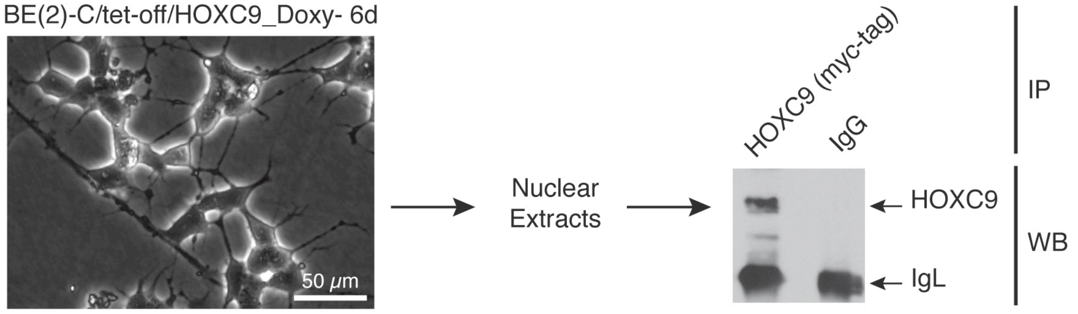

B

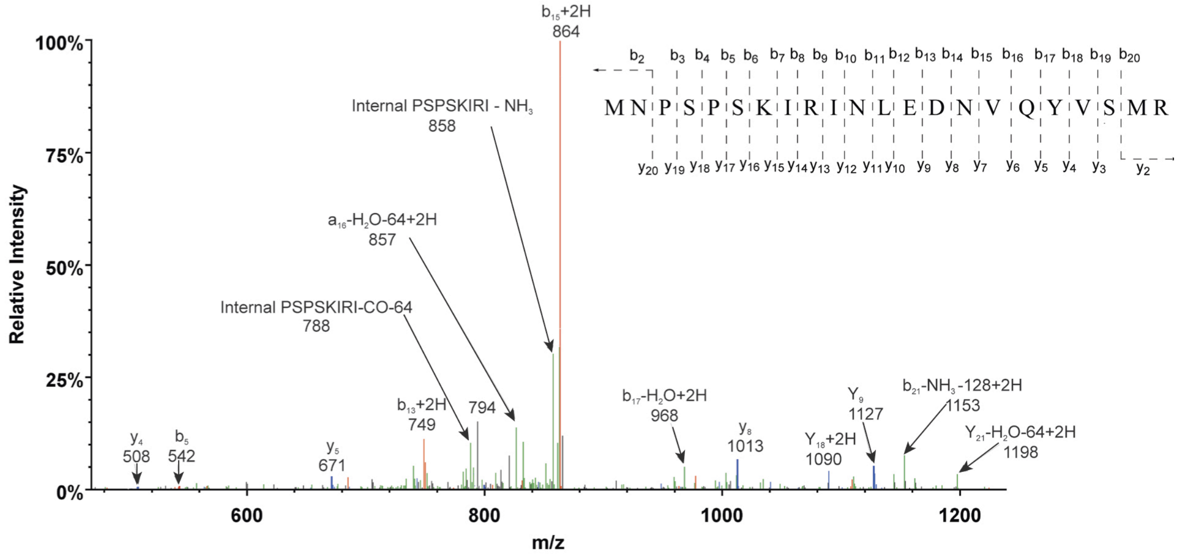

gi|119621336 (94%), 28,102.9 Da
E2F transcription factor 6, isoform CRA_a [Homo sapiens] [MASS=28102]
1 unique peptides, 1 unique spectra, 1 total spectra, 22/249 amino acids (9% coverage)

```
MNPSPSKIRI  NLEDNVQYVS  MRKALKVKRP  RFDVSLVYLT  RKFMDLVRSA  PGGILDLNKV
ATKLGVRKRR  VYDITNVLDG  IDLVEKKSKN  HIRWIGSDLS  NFGAVPQQKK  LQEELSDLSA
MEDALDELIK  DCAQQLFELT  DDKENERLAY  VTYQDIHSIQ  AFHEQIVIAV  KAPAETRLDV
PAPREDSITV  HIRSTNGPID  VYLCEVEQGQ  TSNKRSEGVG  TSSSESTHPE  GPEEEENPQQ
SEELLEVSN
```

C

HOXC9-responsive genes_down
Motif_E2F (n=155)

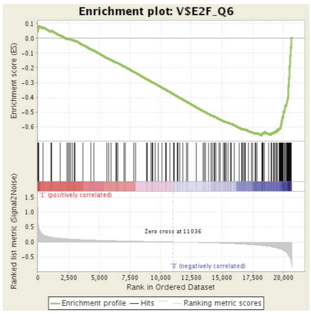

NOM p=0; FDR=0

Figure S3 Identification of E2F6 as a HOXC9-interacting protein. (**A**) Schematic of experiment. Anti-myc-tag immunoprecipitation with nuclear extracts from BE(2)-C/Tet-Off/myc-HOXC9 cells cultured in the absence of doxycycline (Doxy) for 6 days. IP, immunoprecipitation; IB, immunoblotting. (**B**) Collision-induced fragmentation spectra of the E2F6 unique peptide MNPSPSKIRINLEDNVQYVSMR (+3 charge state). Insert shows all possible b and y peptide ions arising from the scission of all available peptide bonds of the E2F6 peptide. m/z, mass-to-charge ratio. (**C**) GSEA showing significant enrichment of E2F-binding motif among the genes downregulated by HOXC9.

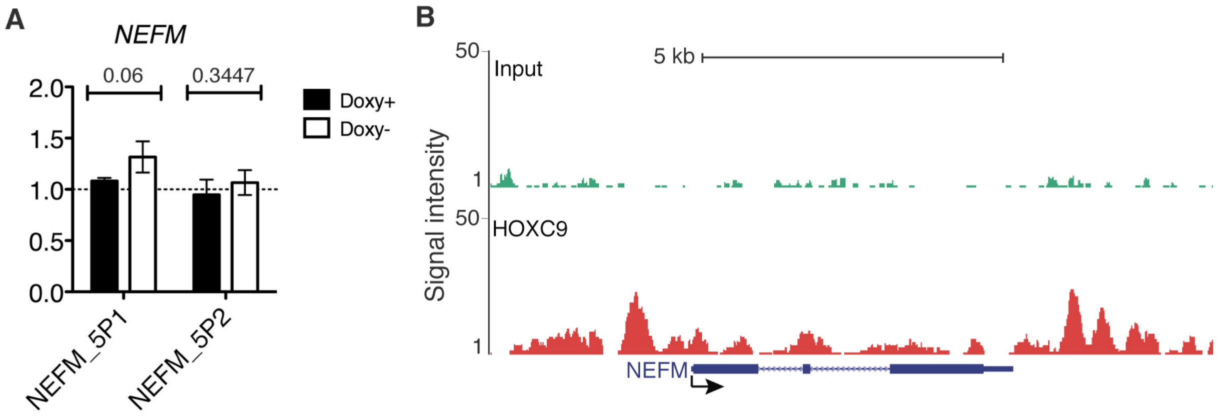

Figure S4 E2F6 does not bind to the neuronal HOXC9 target gene NEFM in vivo. (**A**) ChIP-qPCR analysis showing no E2F6 binding to the promoter of the neuronal gene *NEFM* in BE(2)-C/Tet-Off/myc-HOXC9 cells before (Doxy+) and after (Doxy-) HOXC9 induction. Dashed line indicates IgG control. Error bars represent SD (n = 3). Data were analyzed with unpaired, two-tailed Student's *t*-test and *p* values are indicated. (**B**) ChIP-seq tag profile showing HOXC9 binding to the *NEFM* locus.
